# Similarity based task timely termination method for image based intelligent agents

**DOI:** 10.1038/s41598-024-83463-8

**Published:** 2024-12-30

**Authors:** Sheng Jie, Xing Huang, Chengxi Jing, Xian Jiang, Ligang Dong

**Affiliations:** Zhejiang Gongshang University, SIEE, Hangzhou, 310018 China

**Keywords:** Intelligent agent, Large language model, Application, Task termination, Timely, Mathematics and computing, Computer science, Information technology, Software

## Abstract

Due to the hallucination of the underlying large language model(LLMs) or the unclear description of the task’s ultimate goal, the agents have become somewhat confused. Despite having completed tasks, they have not ceased working, leading to a waste of resource. We propose similarity-based task timely termination method for image-based intelligent agents, This method involves recording the scenario state after the completion of each sub-task and comparing it with the fully completed task scenario state using a structural similarity method. The result is quantified and standardized into a structural similarity index, which is used to judge whether the task has been completed. Moreover, we categorize the types of agents based on model and created an image-based agent task dataset. In experimental results, the image-based agents using this method showed an average reduction of 1.94 steps in the number of steps to complete 20 task tests, a $44.1\%$ reduction in time costs, and a $47.3\%$ reduction in token costs. This method can effectively reduce the negative actions of image-based agents when they experience hallucinations, ensuring their tasks are completed excellently, and it can effectively reduce the waste of resources such as time, tokens, and hardware. Our project can be found at GitHub.

## Introduction

With the release of ChatGPT at the end of 2022, the field of artificial intelligence (AI) has once again welcomed a new wave of enthusiasm. The original ChatGPT supports text conversation; users can input questions through a text box, and ChatGPT can provide satisfactory results based on its extensive training corpus. ChatGPT has opened a new era in the field of AI. As large language model (LLM) technologies, led by GPT, continue to receive attention, a significant amount of funding has also been invested in research in similar fields. In March 2023, GPT-4 was released, adding visual and auditory perception to the capabilities of ChatGPT^1^. Currently, most research based on LLM is established in certain specific domains, fine-tuned through domain-specific datasets, and simultaneously constructed with suitable instruction sets to enhance model performance in specific domains. Among them, the Chain of Thought (COT) method has been proven to effectively improve the model’s reasoning capabilities^2^. For instance, Wang et al.^3^ proposed that self-consistency based on the chain of thoughts has achieved significant improvements in mathematics and commonsense tasks, which can better stimulate the reasoning ability of LLMs and also provide effective technical support for LLMs as core components of intelligent agents.

Based on the form of model input, it can be roughly divided into three categories: text, image, and audio. Currently, a large amount of research is focused on text, using natural language to input questions into the model, and the model’s answers are also in the form of text. For example, Madaan et al.^4^ proposed a method to improve the initial output through iterative feedback and refinement, achieving self-refinement of LLMs; Xi et al.^5^ introduced the concept of self-polish, which encourages the model to progressively refine a given question to make it easier to understand and solve, thereby promoting the model’s problem-solving capabilities; Chen et al.^6^ suggested enhancing the model’s ability to read long documents by processing long contexts into summary node trees and iteratively prompting decisions on how to read the text.

Additionally, research that uses images as input and output formats mainly employs autoregressive methods, converting pixels in images into tokens that can be understood by LLMs. For instance, Huang et al.^7^ proposed the Kosmos model, which can perceive image data and handle various tasks involving visuals; Liu et al.^8^ introduced more high-quality datasets to fine-tune the existing LLaVA models, significantly improving the model’s image recognition and detection capabilities.

The processing of audio is similar to that of images, where audio data is encoded and input into the model, and a transformation mechanism is used to output verbal answers. For example, Zhang et al.^9^ used the ImageBind model, which aligns embeddings of various modalities, combined with an audio Q-former, allowing LLMs to learn sound information; Huang et al.^10^ proposed AudioGPT, which uses ChatGPT as an interface model to integrate audio information into a pre-trained LLM, and the generated answers are converted into voice output through another interface.

Research on images and audio mainly focuses on converting pixels or audio information into tokens that can be understood by the model, to get corresponding format outputs. Among them, vision, as a primary condition for individual sensory information, allows people to obtain a large amount of effective information through images. However, current research, such as the framework proposed by Gani et al.^11^, extracts details such as the layout of images from long texts, applicable scenes, and descriptions of foreground and background objects. By utilizing the reasoning abilities of LLMs combined with diffusion models to generate images, its essence is mainly an extended form of text input, and there is still room for improvement in the understanding of images.

The concept of an agent can be traced back to the field of philosophy, referring to a subject with autonomous consciousness and the ability to act^12^. With the continuous development of science and technology, the concept of AI agents has gradually replaced the original definition of agents, but still retains its connotation. The AI agents we refer to now are those with autonomous capabilities, able to perceive their surroundings and make decisions, and use corresponding tools to take action. The development of agents can be roughly divided into the following three stages. First, there were the reactivity-based symbolic agents, focused on in the 1990s, which mainly concentrated on the interaction between the subject and the environment. They used complex symbols composed of knowledge, combined with expert systems, to simulate human thinking and form clear, highly interpretable reasoning patterns^13^. By the early 21st century, with the further popularization of statistical learning methods, machine learning methods centered around transfer learning and meta-learning were formed. By combining different tasks, corresponding search strategies and loss functions were constructed to improve the accuracy of model reasoning. This also focused on the sharing of knowledge and the learning ability of the agents themselves, enhancing the agents’ generalization capabilities^14^. However, the two aforementioned methods have high requirements for data quality, and agents perform poorly on unknown tasks^15^. At the current stage, LLMs are used as the core part of controlling agents, multimodal perception technology is used to grasp environmental dynamics, and prompt engineering combined with pre-trained corpora serves as the source of agents’ memory, reasoning, and planning abilities^16^. They call APIs and various tools to take action and use feedback mechanisms to learn information about the environment and interact with it^17^.

In the application of agents based on LLMs, their use on mobile phones is of particular interest. Currently, smart voice assistants on mobile phones typically call backend interfaces and built-in function methods to operate applications in response to user queries, requiring the use of advanced system commands, which poses certain security risks to the phone. Therefore, researchers have proposed a method similar to the way humans use mobile phones, that is, to complete tasks by clicking on the screen^18^. This approach interacts directly with the user’s graphical interface, bypassing the system’s underlying logical framework, which simplifies the design and enhances the model’s interpretability, Our experiment is based on intelligent agents operating mobile phones.

Nowadays, existing intelligent agents still have certain issues in judging the end of the task, and cannot end the execution in time when the task has been completed, thus bringing a certain degree of waste to time and economy. In this regard, this paper proposes an effective measure to solve such problems, this method is similarity-based task timely termination method for image-based intelligent agents , by dividing the task into sub-tasks, recording the scene state after the completion of each sub-task and comparing it with the fully completed scene state of the task with the structural similarity method, and obtaining the similarity_index after quantification and standard normalization. Similarity_index, through which the index is compared with the preset satisfaction index, and the comparison result determines the next operation, this paper proposes an effective solution that enables image-based agents to more accurately determine the end conditions of tasks and reasonably reduce resource consumption. Our contributions are summarized as follows:

- We have classified the types of agents based on the base model.
- We have created an image-based agent task dataset, which is applicable to the related work of image-based agents.
- We have established a task timely termination method for agents, allowing agents to find their own task completion markers, reducing unnecessary resource cost wastage.
- Based on the above foundation, we designed and elaborated the similarity-based task timely termination method for image-based agents, and set up a reward feedback mechanism, which to some extent avoids the image-based agents from getting lost and allows them to complete tasks exceptionally well.

## Related work

Agents built on LLMs have been widely applied, yet there remain some unresolved issues. Among these, the hallucination phenomenon of LLMs has long been a vexing problem, which has indirectly caused a certain degree of confusion in agents as well. Hallucination refers to LLMs producing seemingly reasonable but actually nonsensical, false, or unfounded information. This is due to the models being trained on extensive datasets, but the quality of these datasets cannot be effectively guaranteed. They have not been rigorously screened, and the datasets themselves contain a certain amount of erroneous information. This can lead to a degree of knowledge confusion in LLMs, compromising the authenticity and accuracy of the generated information.

Currently, many scholars have noticed this issue and proposed various solutions. From the perspective of model, For example, Xu et al.^19^ introduced the Knowledge-Infused Language Model (KILM), which continuously injects new knowledge into the encoder-decoder to enable the model to retain more knowledge; Wei et al.^2^ proposed the COT method, which simulates the human thought process by breaking down tasks into multiple sub-reasoning tasks, gradually guiding the model to complete the final task, allowing the model to self-improve during reasoning and thereby mitigating the hallucination issue to some extent; Yao et al.^20^ proposed the ReAct paradigm, which explicitly reasons through each step of behavior, allowing the model to self-supervise and calibrate, thus improving the accuracy of the results to a certain extent; Gu et al.^21^ introduced the PPT method that pre-trains prompt words, unifying three types of classification problems into a multiple-choice task, and by pre-training on datasets to obtain related soft prompts, enabling the model to adaptively learn and thus enhance its performance; furthermore, due to the limited length of context and the presence of various errors within it, Xu et al.^22^ proposed the $\textit{k}$NN Prompting method, which uses the $\textit{k}$NN algorithm to find texts related to the input text and adds these texts to the prompt, thereby improving the model’s accuracy and the quality of the output.

Also, some scholars start from the level of intelligent agents, standardizing to improve the accuracy of output results. Recently, intelligent agents have shown significant effects in enhancing the problem-solving capabilities of LLMs. Some research focuses on sociological phenomena, Park et al.^23^ constructed a “virtual town” with 25 agents to explore sociological phenomena such as language interaction, social understanding, and collective memory. Yang et al.^24^ proposed AutoGPT, which innovates at the prompt level, stimulating the model’s capabilities through better prompts. Hong et al.^25^ proposed MetaGPT, innovating at the code level to improve the accuracy and richness of the model’s code generation.Shen et al.^26^ proposed HuggingGPT, whose core concept is to use language as a universal interface between models, enabling LLMs to call external models for complex tasks. Liu et al.^27^ achieved consensus on value judgments and generated an instruction dataset by interacting with LLM agents in a sandbox environment. Through development at the agent level, the potential of the model can be more fully realized, capable of engaging in tasks requiring higher cognitive functions, such as software development by Qian et al.^28^ and Chen et al.^29^, and contributions in gaming by FAIR et al.^30^ and Xu et al.^31^. In the Natural Language Thinking Society (NLSOM), Zhuge et al.^32^ proposed agents with diverse functions to interact through multiple rounds of “mindstorms” to solve complex tasks together. Cai et al.^33^ proposed an innovative model that significantly reduces costs by combining large models as tool manufacturers and small models as tool users. Moreover, many research results have successfully enhanced the performance of LLMs in handling complex tasks by integrating discussions among multiple agents, such as Wang et al.^34^, Du et al.^35^, Hao et al.^36^, and Akata et al.^37^. These studies, starting from the level of intelligent agents, standardize the content of output results, ensuring the integrity of generated information to a certain extent and improving the quality of output.

However, the above methods only help to alleviate the illusion phenomenon to a certain extent and do not fundamentally solve this problem. For the above model-level methods, since the model training process relies on a large amount of data to stimulate the phenomenon of “intelligence emergence”, these methods cannot avoid the illusion problem of LLMs. The confusion problem of intelligent agents is closely related to the illusion problem of LLMs. Therefore, we need to develop corresponding strategies for improvement from the perspective of intelligent agents to solve their confusion problem. However, the strategies described above for intelligent agents only start from the aspect of specialized applications and do not fully consider their universality in solving the model illusion problem, all of which have certain limitations. The confusion problem of intelligent agents based on the architecture of LLMs is a complex task that requires multidimensional research and solutions for finding a way out.

Compared with the above work, the innovations of this paper include (1) This paper proposes the first task timely termination method for agents, which is an effective way to solve the problem by setting the task completion flag at the level of agents to avoid the agents falling into confusion to a certain extent. (2) This paper creates a task dataset of image-based agents, which can be applied to the related work of image-based agents. (3) This paper is based on image-based agents and firstly designs the task termination method for image-based agents. (4) In this paper, a reward feedback mechanism is designed, which can continuously optimize and improve the further operation of the agents, so that the task is completed well enough. All the datasets of this paper and the code of the task termination algorithm for image-based agents can be obtained for free on GitHub(https://github.com/qingqiyangqidanqi/MTTIA).

## Methods

The emergence of agents each has its own specific application scenarios, but the original intent behind each agent is the same, which is to complete a certain type of task, from the “beginning phase” they will ultimately move towards the “completion phase”^38^. However, due to the hallucination issues of the underlying LLMs^39^, or the inaccuracy of task finalization descriptions, agents have also developed a certain degree of confusion. To some extent, agents have already completed the set tasks, but they have not yet ceased working, resulting in unnecessary workload. This significantly leads to the waste of time, hardware, and token resources, among others. To address these issues, this section proposes a task termination method for agents.

### Different categories of agents

**Figure 1:**
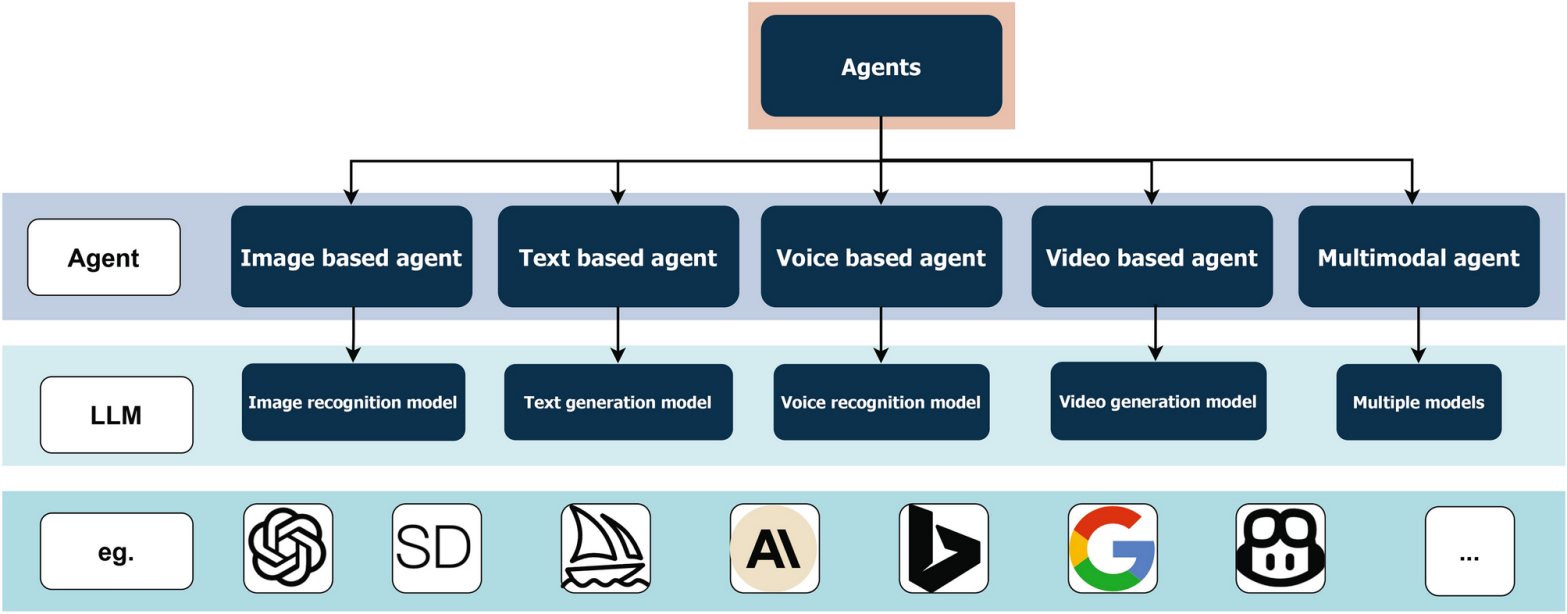
Different Categories of Agents. This image shows different categories of intelligent agent models and their underlying base LLMs.

Nowadays, the common practice in constructing agents is to use different LLMs at the underlying level to complete tasks specific to a certain domain. As shown in Fig. 1, the LLMs used at the base level include four types: image, text, sound, and video. Based on the different LLMs invoked at the underlying level, we can classify agents into five types: image-based agents, text-based agents, sound-based agents, video-based agents, and multimodal agents.

#### Image-based agents

These types of agents can understand and process image-based data by invoking underlying models such as GPT-4V, StableDiffusionXL, Midjourney, etc., and refine the feedback obtained to provide users with better feedback effects. Among them, GPT-4V^40^introduced by OpenAI, is capable of understanding images uploaded by users and generating responses based on the image information, and can transform text to images through DALLE-E3. Users can get images by simply describing the content of the pictures in natural language; StableDiffusionXL model^41^is an image-based model launched by Stability AI, which can produce high-resolution images through text, offering a high-quality visual experience; Midjourney model^42^ developed by Midjourney Lab, can generate AI drawings by describing scenes and styles, and adjusting tones, pixels, and camera language.

Image-based agents rely on models that are pre-trained on image data. These models learn image features, object recognition, scene understanding, and more, primarily using the Convolutional Neural Network (CNN) architecture, such as Residual Network (ResNet) or Efficient Neural Network (EfficientNet). They are suitable for tasks like image classification, object detection, and image generation. In medical image analysis, image-based agents can assist in identifying pathological tissues. In the field of autonomous driving, they are used for environmental perception, recognizing road signs, pedestrians, and other vehicles.

#### Text-based agents

Such agents can understand and process text-based data by invoking underlying models such as chatGPT-4 Turbo, the Claude series, Bing, and Mistral, and use the context space to turn historical QA into memories, ensuring the reasonableness and fluidity of communication. Among them, chatGPT-4 Turbo^43^introduced by OpenAI, is capable of human-like conversational exchanges, showing near-human performance in intent recognition and sentiment analysis; the Claude series models^44^by Anthropic are text-based models that continuously optimize across various task types, thus effectively reducing model hallucinations and increasing dialogue accuracy; Bing Chat^45^is a plugin launched by Microsoft Edge that supports web searches and integrates the required information to produce responsive text outputs; the Mistral model^46^ developed by Mistral AI, enhances the understanding of text-based agents in multiple languages based on text dialogue.

Text-based agents rely on models that are pre-trained on text data. These models learn vocabulary, grammar, and semantics, primarily using the transformer architecture, such as Bidirectional Encoder Representations from Transformers (BERT) or GPT. They are then used for tasks like classification, generation, and more. These models can be fine-tuned to adapt to specific tasks, such as sentiment analysis, question-answering systems, and text generation. Text-based agents can be employed in customer chatbot services, providing quick responses by understanding user queries. They can also be used for content moderation, utilizing LLMs to automatically audit text content on social media and identify inappropriate speech.

#### Audio-based agents

These types of agents can understand and process voice-based data by invoking underlying models such as Google Assistant, Siri, and Cortana^47^, by reflecting on the collected voice data to continually strengthen the model’s capabilities. Among them, Google Assistant controls various applications within its ecosystem and is capable of understanding and recognizing audio signals; Siri is a voice assistant launched by APPLE with basic voice recognition and processing capabilities; Cortana, developed by Microsoft, is an intelligent voice assistant that can also extend the multimodal form of agents.

Audio-based agents rely on models that are pre-trained on audio data. These models learn sound features, sound classification, and more, primarily using architectures such as Recurrent Neural Networks (RNN), Long Short-Term Memory networks (LSTM), or Transformers, like WaveNet or Transformer-TTS. They are suitable for tasks such as speech recognition and sound classification. Audio-based agents can be used in voice assistants, such as Siri or Alexa, where they interpret and respond to users’ voice commands. Additionally, they can be employed for automatic subtitle generation, where audio-based agents convert speech from live presentations or videos into text subtitles.

#### Video-based agents

These types of agents can understand and process video-based data by invoking underlying models such as Sora, Stable Diffusion Video, etc., and continuously collect new data from users or the internet, thereby further enhancing the model’s effectiveness. Among them, Sora^48^is a video generation model released by Open AI, capable of automatically generating up to 60 seconds of high-definition videos based on the information provided by users, with content that closely aligns with existing logic and common sense; Stable Diffusion Video^49^ developed by Stability AI, can transform a series of static images into high-quality video sequences. The emergence of video-based agents marks a significant breakthrough in the field of agents, and AI technology has taken another major step forward.

Video-based agents combine image and audio processing technologies and are pre-trained on video data. They learn motion features, action recognition, video classification, and more, using models such as 3D Convolutional Neural Network (3D CNN), Two-Stream Network, and Video Transformer to process video data. These agents are optimized for tasks such as video classification, event detection, and video generation. Video-based agents can be used for monitoring video streams to identify abnormal behaviors or specific events. They can also be employed for automatic video editing and content generation, such as automatically creating movie trailers.

#### Multimodal agents

These types of agents can understand and process multimodal data such as text, images, voice, and video by invoking underlying models like Copilot, Gemini, etc., and refine the multi-faceted feedback obtained to provide users with more efficient life and work experiences. Copilot^50^, developed by Microsoft, combines the Microsoft ecosystem with smart office concepts, allowing users to issue commands through natural language, images, or voice. Copilot can devise reasonable plans and execute them automatically. Gemini^51^, introduced by Google, supports context inputs of up to 1 million tokens, enabling the analysis of longer texts, audio, and video content, greatly enhancing the model’s memory and understanding capabilities.

Multimodal agents rely on large multimodal models that integrate various data types such as text, images, and audio, achieving cross-modal information fusion and task processing. They primarily use multimodal fusion structures like the Multimodal Transformer, including models such as Vision-and-Language Transformer (ViLT) and UNiversal Image-TExt Representation Learning (UNITER). These agents are designed with multimodal pre-training tasks, such as the Cross-Modal Masked Language Model (CMLM) and Cross-Modal Contrastive Learning (CMCL), making them suitable for tasks like multimodal classification, multimodal retrieval, and multimodal generation.

#### Further expansion of multimodal agents

In real-world scenarios, most tasks are often overly complex, typically involving information that goes beyond simple text and encompasses multimodal content. The next key research direction for agents based on large language models is to master the ability to process and generate multimodal information. This capability will enable agents to perform more powerful functions and is crucial for achieving human-like intelligence levels. Compared to single-modal agents, such as image-based agents, text-based agents, audio-based agents, and video-based agents, multimodal agents face challenges that are more complex and severe.

Today, there are many efficient models or methods for processing multimodal data, with Contrastive Language-Image Pre-training (CLIP) and Flamingo being the most prominent. CLIP^52^is the first model that can be generalized to multiple image classification tasks through zero-shot and few-shot learning. The biggest highlight of CLIP is its ability to map data of different modalities, text, and images, to a shared vector space. This shared multimodal vector space makes tasks like text-to-image and image-to-text much easier. Unlike CLIP, Flamingo can generate text responses. Flamingo^53^ is like CLIP with the addition of a language model, enabling it to generate corresponding text tokens based on the images and text it sees. Multimodal agents built using such models or methods can better accomplish complex tasks in real-life scenarios.

At the same time, there is a range of technologies and algorithms that can enhance the performance of agents in the real world. For instance, the COT^54^ series of algorithms can be fully utilized to further improve the perception, memory, and reasoning capabilities of agents. In terms of perception, whether it’s feedback from the environment or instructions from humans, using COT algorithms allows the agent to gradually receive information, better understand it, and based on this understanding, recognize intentions and convert them into the next task process. Regarding memory, when agents face multiple rounds of interaction with the environment, COT enables them to efficiently store and retrieve long-term memory by iteratively accessing text memory information. In reasoning, by referencing the approach of COT, agents can decompose tasks and make plans and decisions step by step, thereby enhancing the reliability of problem-solving by the agent.

When handling web search tasks, multimodal agents first need to receive the user’s query request as text-based information and use the search bar to retrieve relevant information. Subsequently, they must operate the mouse to browse web pages, examine, and pinpoint key content, which includes extracting key information from online articles, video reports, and social media posts. Following this, they need to process multimodal data, including text, to further integrate content and carry out a series of reasoning steps to obtain the final result, which is then fed back to the user^55^. Although there have been some review studies on LLM-driven agents^38,56,57^, they seldom address the aspect of multimodal processing.

In the early stages of researching multimodal information processing^58–60^, researchers often used basic relevance models or tools to convert image or audio data into text descriptions. However, this approach often resulted in excessive information redundancy, especially when dealing with complex modal content like videos. Recent research progress has turned to utilizing large language models for multimodal agents to handle complex data types. In an open-world gaming environment similar to the real world, Wang et al.^61^ proposes a new method for processing non-textual modal information. This method first acquires visual observation data from the environment, then maps these visual cues along with textual instructions into the GPT model to generate complex action plans. In this way, it achieves planning and control similar to human multimodal observation.

When multimodal agents face unpredictable multi-step user inputs, security becomes a major challenge^62^. Users play a significant role in interacting with agents, influencing the direction and outcome of the agent’s execution through multiple rounds of feedback. However, these multi-step inputs also pose challenges. Inadequate content in user inputs can affect task outcomes. Moreover, there is the risk of malicious users intentionally guiding the agent to execute unsafe code or operations. Therefore, it is necessary to design highly flexible agent ecosystems that can understand and adapt to the diversity of user inputs while ensuring robust security measures to prevent malicious activities and misleading user inputs.

### Task timely termination method for agents

**Figure 2:**
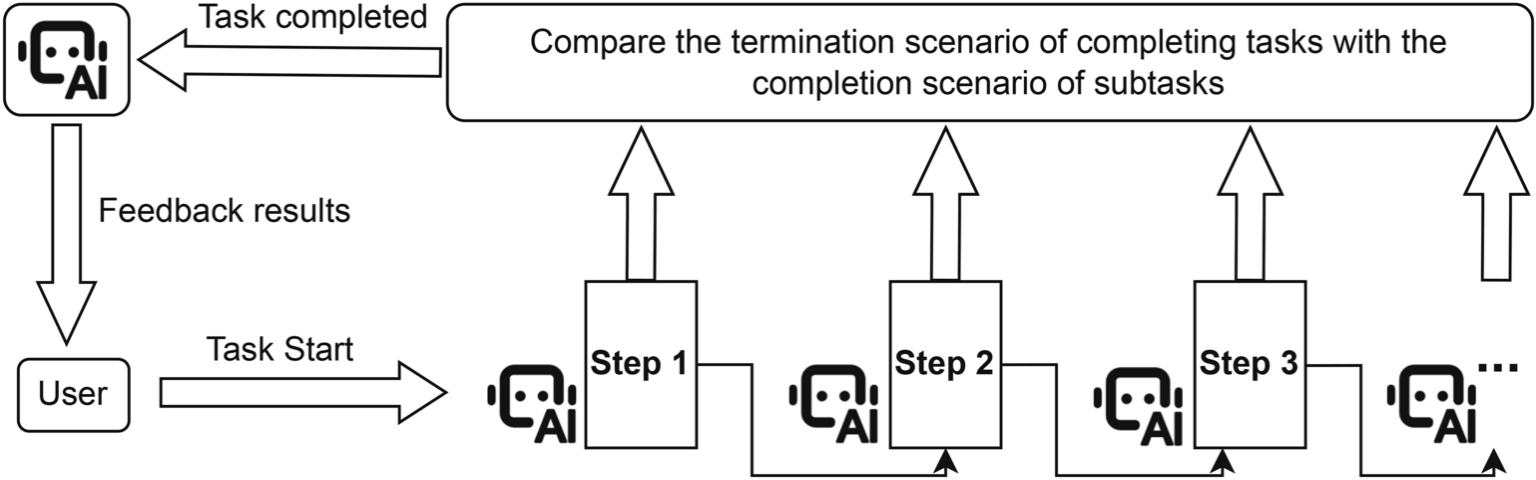
Method flow for timely Terminating Tasks of Agents. This diagram illustrates the entire process of the agent’s task termination method, in which the agent completes the task in an orderly subtask, compares the situation each time, determines the task to be completed when the conditions are met, and feeds back the result to the user.

The method of terminating an agent’s task involves setting a termination pattern that allows the agent to identify its own task completion signals while ensuring the task is completed excellently. As shown in Fig. 2, specifically, when a user assigns a specific task to the agent, the task will be divided into many ordered subtasks according to its logical sequence. Only when all subtasks are completed does it mean that the agent’s task is completed. The agent will be given a task completion termination scenario in advance. The agent uses the task termination method to compare the task completion termination scenario with the subtask completion scenario, quantify it, and standardize it to obtain a specific parameter result. We will set a threshold before the agent executes the task. The agent will compare the specific parameter with the threshold, and the judgment criterion at this time is as shown in Equation 1:

1 $$\begin{aligned} Result=\left\{ \begin{matrix} Completed\ excellently & \frac{Parameters}{Threshold}>1 \\ Completed\ basically & \frac{Parameters}{Threshold}=1 \\ Not\ completed & \frac{Parameters}{Threshold}<1 \\ \end{matrix} \right. \end{aligned}$$

### Similarity-based task timely termination method for image-based intelligent agents

**Figure 3:**
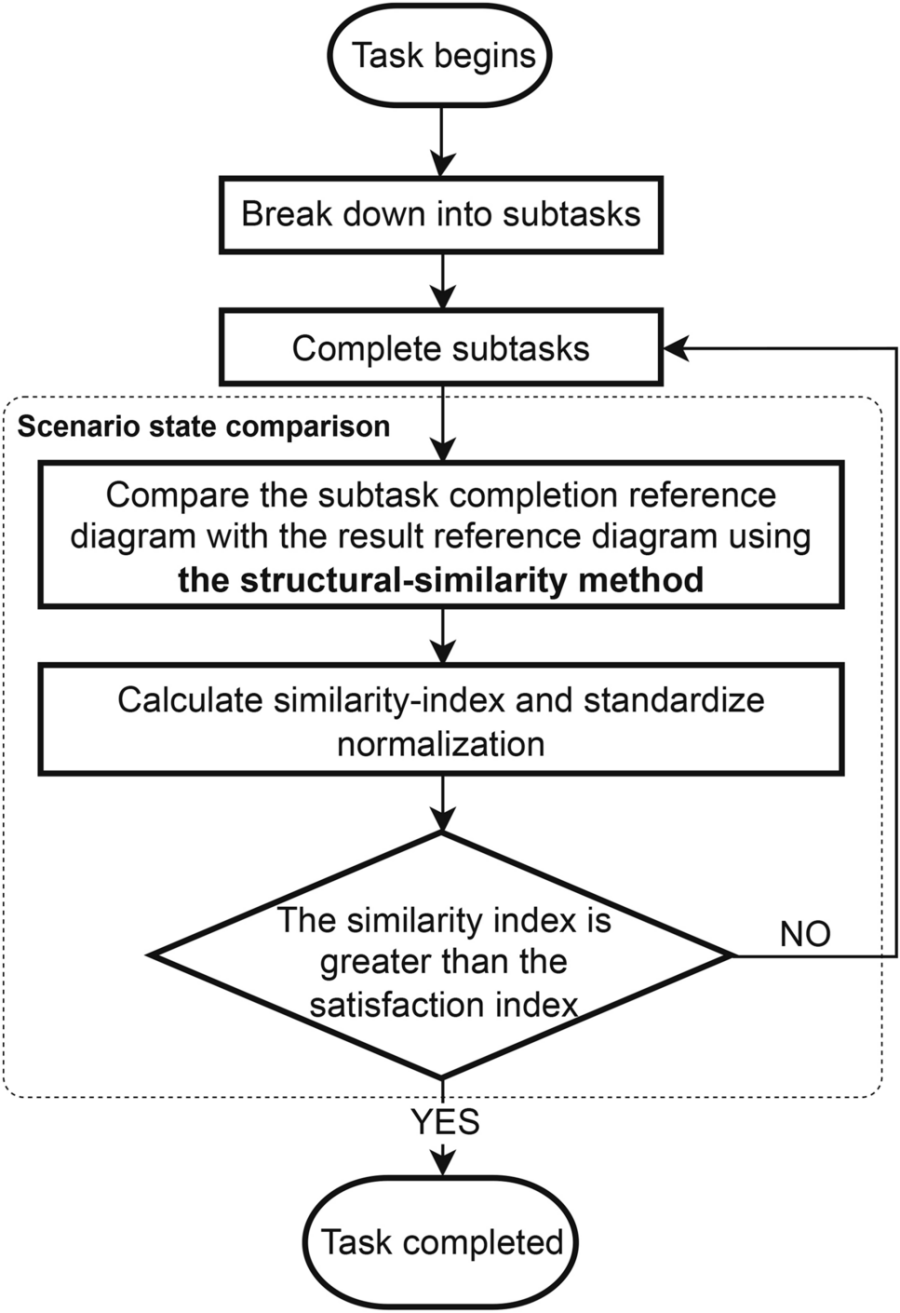
Similarity-based task timely termination method for image-based intelligent agents. This image illustrates the entire process of task termination for image-based intelligent agents.

Building on section , the method of task termination for image-based agents, an image-based agent will pre-set a result reference image, which represents the scene state when the agent has completed the last subtask. As shown in Fig. 3, when the agent completes each subtask, a subtask completion reference image is generated. The agent will process these two images concurrently, as demonstrated in Eqs. 2, 3 and 4. The two images are quantified and standardized through the Structural Similarity method to calculate the similarity_index of these two images. The Structural Similarity is a method for evaluating the similarity between two images, considering brightness, contrast, and structural information of the images. The calculating structural similarity is shown in Equations 2, where $\mu _1$ and $\mu _2$ represent the brightness information of the images. $\sigma _1^2$ and $\sigma _2^2$ represent the contrast information of the images. $\sigma _{12}$ represents the structural information of the images. $c_1$ and $c_2$ are constants introduced to avoid division by zero, typically set to very small positive numbers.

2 $$\begin{aligned} {\begin{matrix} temp\_var& =\operatorname {StructuralSimiilarity}(img1,img2)\\ & = \frac{(2\mu _1\mu _2 + c_1)(2\sigma _{12} + c_2)}{(\mu _1^2 + \mu _2^2 + c_1)(\sigma _1^2 + \sigma _2^2 + c_2)} \end{matrix}} \end{aligned}$$

3 $$\begin{aligned} similarity\_index=\operatorname {StandardScaler}(temp\_var) \end{aligned}$$

4 $$\begin{aligned} satisfaction\_index=\alpha \left( k_1 \beta + k_2 (1 - \beta ) \right) + \left( 1 - e^{-Reward} \right) \end{aligned}$$

We will pre-set a satisfaction_index, which can also be referred to as a threshold which is used to judge whether the task has been completed. Determine the size of the satisfaction_index through quantitative analysis. We introduce the user level of importance $\alpha$, the page layout diversity index $\beta$, and the reward for similar tasks performed previously, as well as the coefficients $k_1=0.62$ and $k_2=0.77$. For further details on the specific analysis process for setting the $k_1$ and $k_2$ coefficients, please refer to section . By comparing the structural similarity index, with the pre-set satisfaction index, the judgment criterion at this time is as shown in Equation 5:

5 $$\begin{aligned} Result=\left\{ \begin{matrix} Completed\ excellently & \frac{similarity\_index}{satisfaction\_index}>1 \\ Completed\ basically & \frac{similarity\_index}{satisfaction\_index}=1 \\ Not\ completed & \frac{similarity\_index}{satisfaction\_index}<1 \\ \end{matrix} \right. \end{aligned}$$

For the final judgment result, the agent will invoke a feedback adjustment mechanism to improve its future performance. According to cybernetics and control theory, feedback refers to the mechanism by which a system’s output is returned as input to the system, causing the output direction to change in order to maintain the system’s relative stability. The agent adjusts its subsequent behavior based on the judgment of the task completion results^25^, maintaining relative stability in its actions, progressing step by step towards the completion of the goal, and avoiding confusion. Specifically, it will make some events in response; when the task is determined to be completed excellently, the agent will give itself a substantial reward; when the task is judged to be just basically completed, the agent will give itself an appropriate reward; when the task has not reached the anticipated level, the agent will judge the task as incomplete and, to encourage itself, will give itself a small reward. In the early stages of task execution by agents, if a similar task has been performed previously with a high reward score, the agent will significantly rely on the previous work to execute the task, this approach reduces the need for structural similarity methods, thereby accelerating task processing and reducing computational resource usage. Conversely, if the agent has not previously encountered a similar task or has done with a low reward score, it will employ structural similarity methods to enhance task completion rates.

This method eliminates the negative actions that image-based agents might take when they are confused. Negative steps refer to the image-based agents not having a definite predetermined conception and expectation for success in the task completion, and engaging in ineffective redundant steps that do not help the task at all. More importantly, this method significantly reduces the consumption of resources such as time, hardware, and tokens.

## Results

In this section, we evaluate and analyze the effectiveness of the similarity-based task timely termination method for image-based intelligent agents through a combination of quantitative and qualitative experiments. Our primary evaluation objective is the effective suppression of unnecessary resource cost consumption by the image-based agents’ task termination methods.

### Experimental environment

In this section, we will describe the detailed information of this experiment. The intelligent agent tested in our experiment is AppAgent developed by Tencent, and all tasks are executed on an Android 12 virtual machine. The hardware foundation for running the intelligent agent code is a standard desktop computer equipped with an Intel i5 processor and a GeForce GTX 4060ti graphics card, with the operating system being Windows 11 Professional Edition. Unless otherwise specified, we use gpt-4-vision-preview as the model to determine the decisions of the intelligent agent in our experiments. At the same time, to ensure that the intelligent agent does not run indefinitely during the experiment, we set the maximum number of task completion rounds for the agent to 20. To ensure the cost-effectiveness of the experiment and the validity of the execution steps, we set the maximum number of tokens per round of task execution to 300, and the time between consecutive GPT-4V model requests to 5 seconds. The following is the data required during the experiment, as well as the content of the evaluation metrics for the experiment.

#### Data

In this section the data used during the experiment will be described in detail. The task dataset we created for image-based agents is applicable to related work. The task dataset includes data for routine operations on mobile applications and can be divided into two types of task data: tasks with unified page layouts and tasks with frequently changing page layouts. Each task data specifically includes mobile Applications, operational task description, task result reference image, and triple objects.Specifically, the dataset includes 20 apps such as Clock, Chrome, and X, with 10 tasks set for each app. The execution steps for each task do not exceed 20 steps. The tasks include specific actions such as “Set an alarm at 6:15 am every Tuesday,” “Search for Zhejiang Gongshang University,” and “Follow musk on x,” among others. Additionally, some tasks are accompanied by result reference images to help the intelligent agent better determine whether the task has been completed.All of the above datasets are available on the GitHub (https://github.com/qingqiyangqidanqi/TTMI_for_Agent) for others to use.

**Mobile Applications:** Our experiment is based on the Android mobile environment, where we have selected several typical Android mobile applications as the subjects of our experiment, including X, Google Map, Gmail, Discord, YouTube, Twitch, Wikipad, Chrome, Excel.

**Operational Tasks:** We have constructed a simple task dataset and made it public. This dataset is based on the aforementioned mobile applications and describes some routine human affairs tasks, which we have described in natural language. Here’s a reference example: “X: Follow musk on X.”

**Result Reference Images:** The result reference image refers to the final result reference image set for a specific task when a particular mobile application is used as the application scenario.

**Triplet Objects:** The triplet object refers to the triplet sequence (mobile application, task, result reference image). By searching for keywords related to the mobile application and task, the corresponding result reference image can be located and invoked.

#### Evaluation metrics

In this section the relevant evaluation metrics of the experimental results will be described in detail.

**1. Completion Rate: **Completion rate refers to the proportion of tasks completed when the similarity index is greater than the satisfaction index.

**2**. **Correct Completion Rate**: The correct completion rate refers to the proportion of tasks that are correctly judged as completed in the total completed tasks, as there are some tasks that are judged as completed but are actually not completed.

**3. Total Task Completion Steps: **The number of completion steps refers to how many steps an agent needs to take to complete a task within a specific application.

**4. Average Task Completion Steps**: The average number of completion steps refers to the average number of steps required for an agent to successfully complete a task within a specific application.

**5. Time Cost:** The time cost refers to the duration an agent takes to complete each task. This evaluation metric can vary due to differences in actual experimental conditions, such as different computer environments or the use of different LLMs, which can cause variations.

**6. Token Cost:** The token cost refers to the total number of tokens consumed by the agent to call all LLMs upon completion of each task. Since tokens need to be purchased with money, token cost often symbolizes the economic cost.

**7. Rewards:** Rewards are given to encourage agents to work more diligently and can also be used to assess their performance. Specifically, a task can be divided into multiple steps, and the closer each step is to the final goal upon completion, the more rewards are given. Even if the task is not completed, a certain reward is still given as encouragement to prevent the agent from entering a state of confusion.

### Baseline

In order to more comprehensively evaluate the similarity-based task timely termination method for image-based intelligent agents, we considered the effects of different environments on their effectiveness and reliability. Therefore, we conducted this experiment under different hardware and software configurations to provide a reasonable and reliable set of experimental results. The agent we tested in this experiment is Tencent’s AppAgent. We set it up with and without the use of the similarity-based task timely termination method for image-based intelligent agents and tested and recorded relevant data during the experiment for a comprehensive comparison.

### Results and analysis

During the course of this experiment, we conducted comparative tests on AppAgent agents with and without the use of the image-based agent task termination methods. We tested 20 basic operational tasks, all of which came from the image-based agent task dataset mentioned in section 4.1. The test results demonstrated the effectiveness of the image-based agent termination methods.

**Figure 4:**
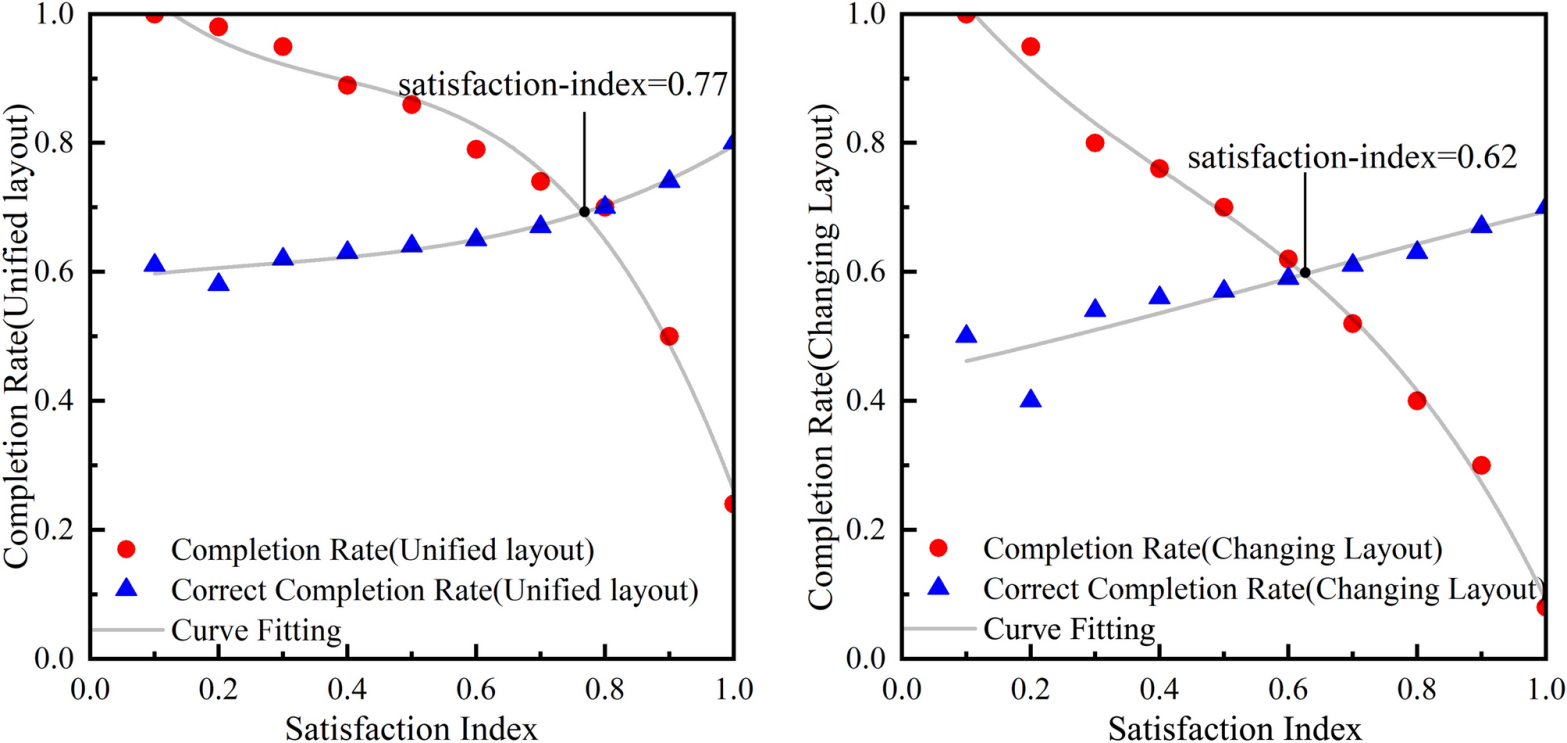
Experimental results of satisfaction index setting. The left graph tests the task of unifying page layout, while the right graph tests the task of frequently changing page layout. Changing the satisfaction index will cause fluctuations in completion rate and correct completion rate. We fit the measured scatter data with a third-order polynomial curve and obtain two intersecting curves.

When evaluating the similarity index of two images, the setting of the satisfaction index needs to be determined based on the specific task scenario. We divided the 20 tasks into two categories and tested the completion rate and correct completion rate of the tasks separately. The first category is the task of uniform page layout, and there will be no significant deviation when executing the task again, such as operating a phone to turn on a flashlight; The second type is tasks with frequent changes in page layout, and there is a certain deviation when each task is completed, such as completing online shopping checkout, where the product interface is different each time. The experimental results are shown in Fig.4. When facing the task of uniform page layout, a high satisfaction index is required for similarity detection while ensuring completion rate and correct completion rate. When the page layout of a task frequently changes, the similarity satisfaction index can be appropriately reduced while ensuring the completion rate and correct completion rate. When considering two specific types of task scenarios, the value of the satisfaction index will also change, as detailed in section .

**Figure 5:**
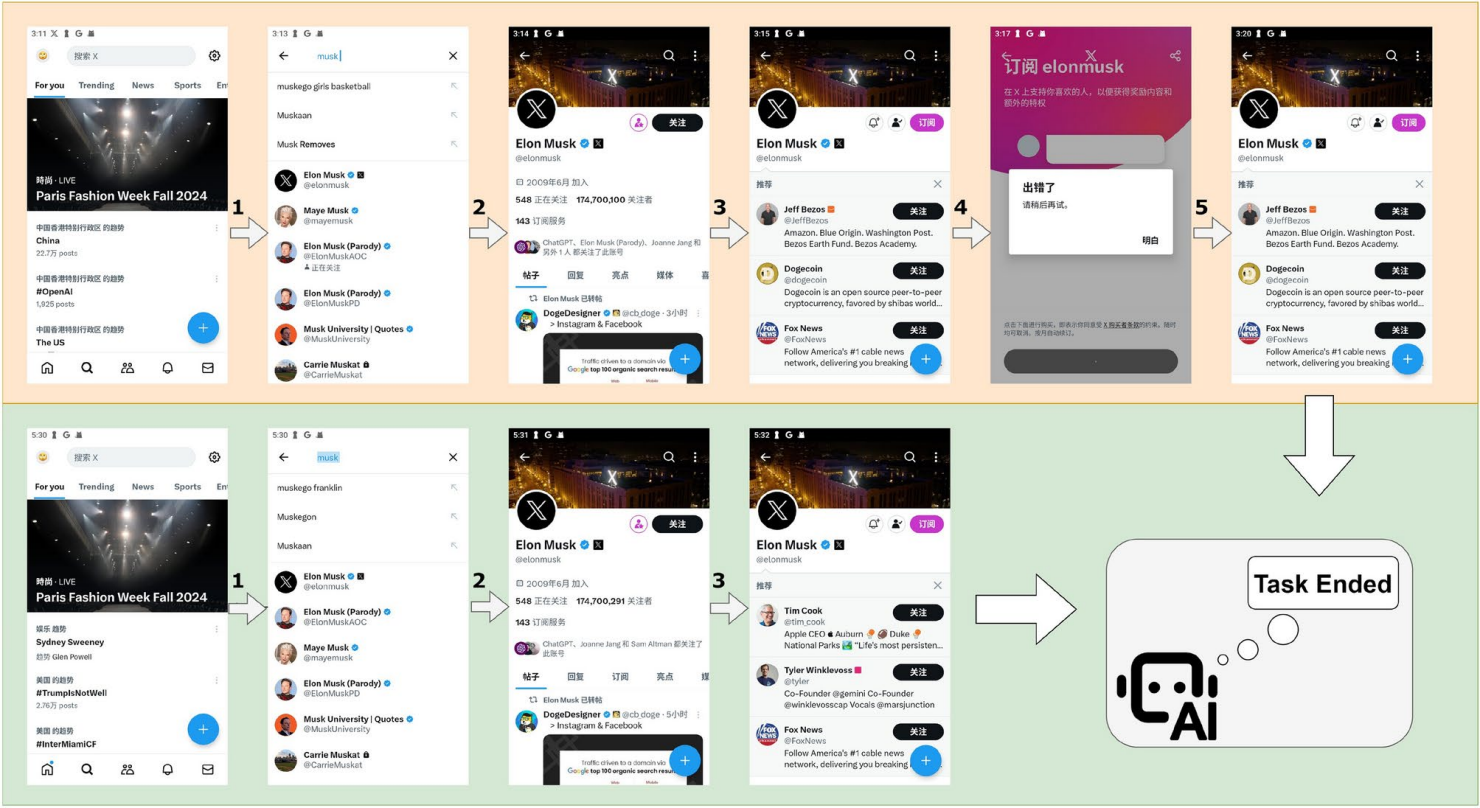
Comparison of steps with and without the image-based agent task termination method. The upper part of the graph details the steps taken by the AppAgent when executing the task without using the method, while the lower part details the steps taken by the AppAgent when executing the task with the use of the method.

Taking one of the test cases as an example for specific analysis, the test application is “X,” and the task is “Follow Musk on X.” The AppAgent, without using the image-based agent task termination method, required 5 steps to execute the task, with the third step already completing the task. However, due to the agent’s illusion problem, 2 unnecessary steps were taken. After employing the image-based agent task termination method, the AppAgent compares the result image generated after each step with the reference result image, and calculates the structural similarity index between the two images. Once the third step is completed, and the image similarity index exceeds the satisfaction index, we can determine that the task has actually been completed. The experimental results are shown in Fig. 5. The similarity-based task timely termination method for image-based intelligent agents can prevent unnecessary redundant steps of the AppAgent from occurring, thereby alleviating the illusion problem of the image-based agents to a certain extent.

**Figure 6:**
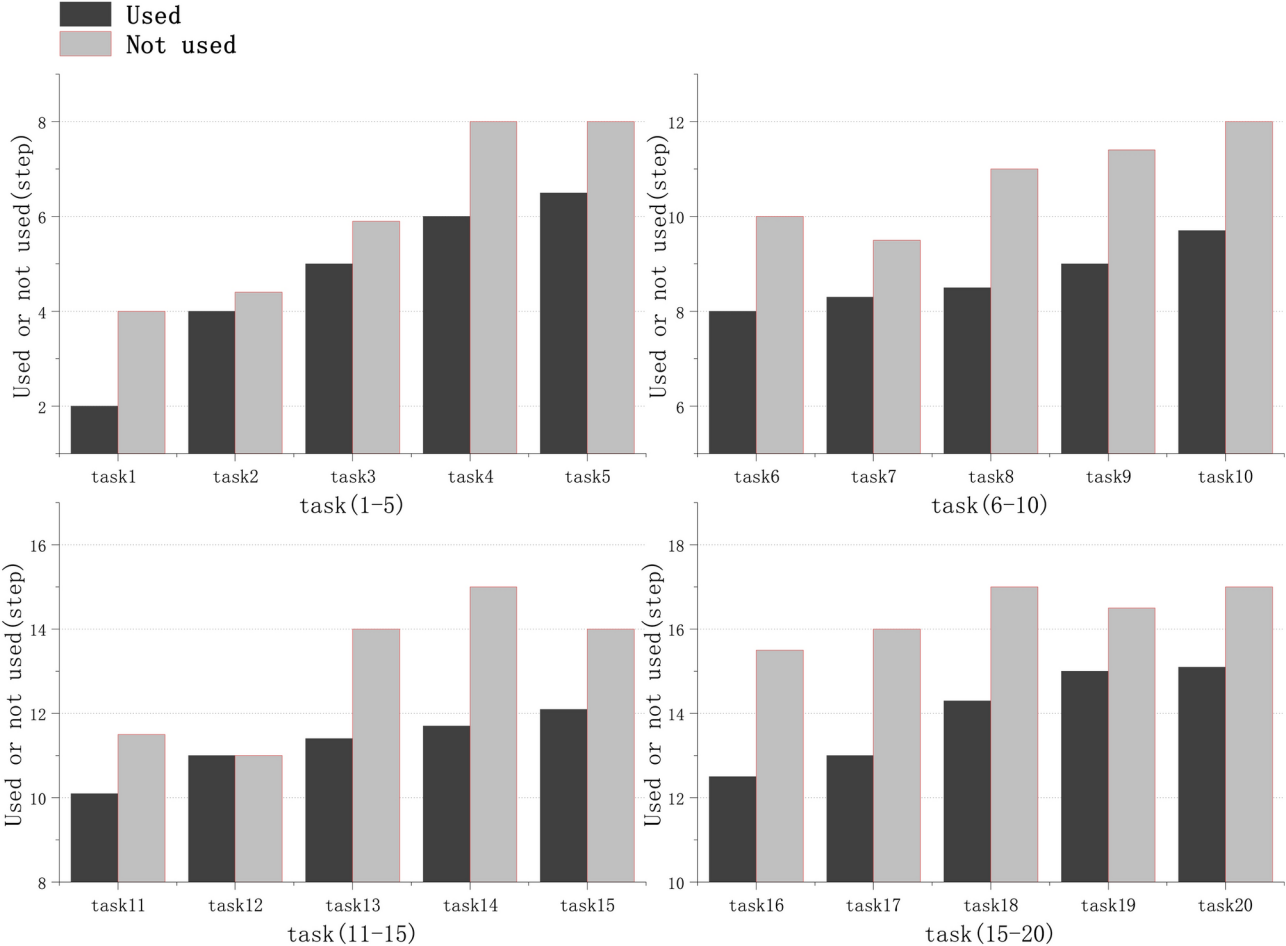
The total number of steps completed in each of the 20 task data. We tested the total number of steps for tasks with and without the use of the image-based agent task termination method across 20 datasets. In our test results, the total number of completion steps for tasks that utilized the method was consistently lower than that for tasks that did not use the method.

We conducted experiments on the number of steps required to complete tasks under consistent experimental conditions, which can be referred to in the section on the experimental environment. We first recorded the total number of completion steps for each of the 20 basic operational tasks executed by the AppAgent without the use of the image-based agent task termination method. Afterward, we applied the image-based agent task termination method within the task execution flow and recorded the total number of completion steps for each of these 20 basic operational tasks. We compared and presented the data results in the form of a bar chart, and the experimental results are shown in Fig. 6. The results indicate that the total number of completion steps for tasks that used the method was consistently less than for those that did not use the method. Therefore, our experimental results prove that the similarity-based task timely termination method for image-based intelligent agents can effectively reduce unnecessary redundant steps taken by image-based agents.

**Table 1:** Task completion with and without the image-like agent task termination method. The AppAgent that utilized the method had a lower average number of steps to complete tasks, higher efficiency, and consumed less in terms of time and token costs. This indicates that the method can effectively reduce the consumption of resources.

**Evaluation metrics**	**Whether to use the method (means±s.e.m.)**		**t**	**p (significant)**
	**Used (n=20)**	**Not used (n=20)**		
Number of steps completed	9.66±0.82	11.6±0.91	$|t|=9.952>t_{crit}$	$<0.05$
Time (min)	3.46±0.30	6.19±0.51	$|t|=11.006>t_{crit}$	$<0.05$
Token	2029.35±183.47	3848.20±300.73	$|t|=14.283>t_{crit}$	$<0.05$

We conducted a statistical analysis test, and under the condition of a significance level of $p=0.05$, the data was found to follow a normal distribution with equal overall variance. A paired sample t-test was performed, and the results indicated that there were significant differences in performance indicators before and after applying this method across in tasks.The experimental results are shown in Table 1, AppAgent performed 20 basic operational tasks, in which the average number of completion steps decreased by 1.94 steps, the time cost was reduced by $44.1\%$, and the token cost was reduced by $47.3\%$. The results indicate that the use of the method resulted in fewer average completion steps, shorter time consumption, and less token consumption. Therefore, our experiment proves that the similarity-based task timely termination method for image-based intelligent agents can effectively prevent agents from entering a state of confusion, ensure tasks are completed to a high standard, and reduce unnecessary waste of resource costs.

Although this method can reduce resource cost wastage, it requires a certain amount of computational resources to perform similarity comparisons, especially when a large amount of information needs to be fed back to the LLM, resulting in higher token costs. For tasks with fewer execution steps and shorter durations, the computational overhead from similarity comparison may be less than the benefits of resource savings. However, for tasks with many execution steps and longer durations, a similarity comparison is required each time a subtask is completed, which can increase computational costs as the number of subtasks grows. In such cases, the computational overhead from using this method may exceed the benefits of resource savings. To address this, it is necessary to consider factors such as the number of execution steps and the risk level of the task. For tasks with long execution steps and high importance, priority should be given to using the similarity-based task timely termination method for image-based intelligent agent, even if it means incurring additional computational costs. On the other hand, for tasks with shorter execution steps and moderate importance, the method can be used only at the final completion of the task to reduce computational costs.

Why is the task termination method for image-based agents effective? In many practical situations, agents need to have a planned, step-by-step approach to solve a specific problem. However, things are always changing, and simply following the initial plan often leads to failure. The similarity-based task timely termination method continuously reminds the agent during its working phase and sets a final goal as motivation. When the agent’s progress deviates towards the wrong direction, it can iteratively make and modify plans based on the feedback reward mechanism, allowing it to proceed methodically towards the goal. Specifically, this method uses a reference image of the subtask completion results during the intermediate process to determine the completion status of the intermediate results. If the similarity index of the subtask does not reach the pre-set satisfaction index, the result is deemed incomplete. The execution process is then retained as an experience document for feedback, and the agent is directed to return to the previous step to re-execute. Subsequently, when the agent uses a large model for task planning, it will avoid the erroneous steps documented in the experience document. This process ensures that the agent does not repeat the wrong approach. Moreover, we have set a maximum limit of 20 task completion rounds for the agent, preventing it from repeating meaningless actions. Based on the experimental results, we introduced page layout diversity index and other factors to adjust the satisfaction index. Under these conditions, the average number of steps to complete the task decreased from 11.6 steps to 9.66 steps, and the average execution time decreased from 6.19 minutes to 3.46 minutes, showing a significant improvement in efficiency.

For rare special situations, when there is not much difference between the intermediate situation of the task and the reference image of the final completion of the task, there may be situations where the task is not completed but is judged to end early. In response to such situations, we introduce a user feedback mechanism. When the execution steps and completion time of a task are far below the average level, we remind the user and ask them to evaluate the completion status of the intelligent agent’s task. Based on this, we adjust the result reference images of each stage of the task and the parameters of the similarity-based task timely termination method for image-based intelligent agent.

Applying the similarity-based task timely termination method for image-based intelligent agent to real-world scenarios can play a significant role in various industries and fields. Nowadays, smartphones have become an essential part of people’s lives, constantly requiring the processing of a large amount of information. Designing an efficient and reasonable smartphone intelligent assistant can significantly enhance user experience and efficiency. A smartphone intelligent assistant that incorporates the image-based intelligent agent task termination method can determine whether a task is completed based on a preset similarity threshold, thereby reducing misoperations and errors. It can also continuously optimize its behavior based on user feedback, thus improving the accuracy of task completion. In today’s life, robots can perform various tasks, such as moving objects, cleaning rooms, and accompanying the elderly. After using the image-based intelligent agent termination method, robots embedded with intelligent agents can better judge whether a task has been successfully completed. For example, in the task of moving objects, the intermediate movement process and final position of the robot can be recorded. After the intelligent agent relies on LLM for thinking, it performs a similarity comparison. If the similarity reaches the set threshold, the task is deemed complete. This method can help robots complete various tasks more effectively and improve their autonomy and reliability. The similarity-based task timely termination method for image-based intelligent agent can be applied to various real-world scenarios and play a significant role in multiple industries and fields.

Overall, our image-based agent task termination method effectively mitigates the state of confusion in image-based agents, avoiding redundant and ineffective steps. It ensures that the agent can complete tasks to a high standard. Notably, our method significantly reduces the consumption of excess resources, such as time, hardware, and tokens, thereby greatly reducing costs. The experiments and analysis presented in this section validate the effectiveness of the method.

## Discussion

This paper introduces a similarity-based task timely termination method for image-based intelligent agents. This method involves recording the scene state after each subtask is completed and comparing it with the fully completed task state using the Structural Similarity method (StructuralSimiilarity), quantifying and normalizing it into a structural similarity index (similarity_index). This index is used to judge whether the task has been completed by comparing it with a pre-set satisfaction index. If the conditions are met, the agent will determine the task as completed; otherwise, it will continue the task and provide appropriate encouragement. In our experiments, this method effectively prevents negative actions by image-based agents when experiencing illusions, ensuring tasks are completed to a high standard. More importantly, this method effectively reduces the waste of resources such as time, tokens, and hardware costs. Additionally, we have categorized the types of agents based on the base model, and we have created an image-based agent task dataset, which can be applied to related work with image-based agents.Some future work directions may be proposed.

Some future work directions may be proposed. Firstly, improving cross-modal applications, we will explore methods for timely termination of tasks for the other four types of agents. Secondly, a reasonable satisfaction index, the satisfaction index for image-based intelligent agents is set based solely on the differences in task page layout, we will customize the satisfaction index according to different application scenarios. Thirdly, reducing the impact of image quality, such as resolution and lighting, may affect this method due to image quality issues, we consider introducing image preprocessing steps to enhance the method’s adaptability and robustness to images of varying quality. Fourthly, adapting to dynamic environments, tasks with dynamic elements will have higher demands on the accuracy of the method, we will explore termination methods suitable for dynamic environments.

## Data Availability

The data used to support the findings of this study are available upon request from the corresponding authors.
